# Study of the Osteoimmunomodulatory Properties of Curcumin-Modified Copper-Bearing Titanium

**DOI:** 10.3390/molecules27103205

**Published:** 2022-05-17

**Authors:** Danhong Chen, Chengcheng Yu, Ying Ying, Yuanyi Luo, Ling Ren, Caizhen Zhu, Ke Yang, Buling Wu, Qi Liu

**Affiliations:** 1 Department of Stomatology, Nanfang Hospital, Southern Medical University, No. 1838 North Guangzhou Avenue, Guangzhou 510515, China; loft163126@163.com (D.C.); jamesy214@163.com (C.Y.); galaxyyying@163.com (Y.Y.); 2 College of Stomatology, Southern Medical University, No. 1838 North Guangzhou Avenue, Guangzhou 510515, China; 3 Institute of Low-dimensional Materials Genome Initiative, College of Chemistry and Environmental Engineering, Shenzhen University, Shenzhen 518060, China; luoyuanyilyy@163.com (Y.L.); czzhu@szu.edu.cn (C.Z.); 4 Institute of Metal Research, Chinese Academy of Sciences, Shenyang 110016, China; lren@imr.ac.cn (L.R.); kyang@imr.ac.cn (K.Y.)

**Keywords:** curcumin, copper-bearing titanium, bone immunoregulation, surface modification, dental implant

## Abstract

Peri-implantitis can lead to implant failure. In this study, curcumin (CUR) was modified onto the copper-bearing titanium alloy (Cu-Ti) with the assistance of polydopamine (PDA) in order to study the bone immune response and subsequent osteogenesis. FE-SEM, XPS and water contact angle were utilized to characterize the coating surface. Bone marrow mesenchymal stem cells (BMSCs) and macrophages were cultured separately and together onto the CUR modified Cu-Ti. Cell activity, expression of relative genes and proteins, cell migration ability, and fluorescence staining of cells were performed. CUR modification slightly increased the activation of M1-type and M2-type cells under physiological conditions. In the inflammation state, CUR inhibited the overexpression of M1 macrophages and induced M2-type differentiation. In addition, the modification itself could provoke the expression of osteoblastic-related genes of BMSCs, while promoting the osteogenic differentiation of BMSCs through the activation of macrophages in both physiological and inflammatory states. The BMSCs migration was increased, the expression of osteogenic-related genes and proteins was up-regulated, and alkaline phosphatase activity (ALP) was increased. Thus, the modification of CUR can promote the osteointegration effect of Cu-Ti by bone immunomodulation and may, in addition, improve the success rate of implants.

## 1. Introduction

After dental implantation, peri-implantitis is often caused by bacterial infection, leading to implant failure [1]. In response, researchers have attempted to modify the implant materials, such as adding a copper element to pure titanium to create a synthesized alloy with an antibacterial function [2,3,4,5]. Moreover, the implant surface has been modified by physical or chemical means to optimize the implant surface topography, or by applying modification with covalent immobilization bioactivate molecules in order to obtain antibacterial properties [6], while promoting the osteogenesis effect of bone marrow mesenchymal stem cells (BMSCs) on implant surfaces [7].

Implant materials with an antibacterial function and the ability of facilitate bone regeneration appear to be able to reduce postoperative complications and maintain long-term stability. However, the immune response of the host and inflammatory factors produced by immune cells play a more pertinent role [8,9]. The immune regulation process of the host primarily involves macrophages, whereby the original macrophages can differentiate into M1-type inflammation-associated cells or M2-type anti-inflammatory cells according to the body’s response [8,10,11,12,13]. Through the response of macrophages, the differentiation behavior of BMSCs is regulated to determine the post-implantation osteogenic effect [12]. 

Metallic titanium, as the most broadly employed material in oral bone repair due to its predominant mechanical and osseointegration properties, could lead to alveolar bone being directly attached with less fibrous tissue formation [14]. Unfortunately, the osteoimmunomodulatory and anti-infection capacities of pure titanium are not outstanding [15]. Therefore, modifications of titanium with new biomaterials were popularly promoted to achieve better biological behaviors. Those new biomaterials generally could be divided into metal material with outstanding mechanical and osseointegration properties, and nonmetallic materials with very good biodegradability and biocompatibility [16]. 

Copper-bearing titanium alloy (Cu-Ti) is a newly developed implant material that can effectively inhibit bacteria and promote the osteogenic differentiation of BMCSs [2,3,4]. However, its response in the immunomodulatory process has not been explored to date. Additionally, other research has revealed the pro-inflammatory risk of the Cu ion [17]. Moreover, peri-implantitis is not only associated with bacterial infection and bone resorption, but also with excessive fibrous encapsulation around implants [18]. In order to further improve the immune regulation effect of Cu-Ti, we have identified a cheap and widely available nonmetallic chemical that has the aforementioned benefits, curcumin (CUR), an important component derived from turmeric that has multiple effects such as anti-inflammatory, anti-bacterial and anti-tumor, as well as the inhibition of bone resorption and fibrosis [19,20]. In addition, CUR can inhibit the M1-orientation differentiation of macrophages [21,22], induce the M2-orientation differentiation of macrophages [23], or transform M1-type cells into an M2-orientation [11,24]. Moreover, CUR has been proven to promote the osteogenic differentiation of BMSCs through the immune response of macrophages [25]. Therefore, we hypothesize that CUR modified on the surface of copper titanium metal could have positive effects on bone immune regulation and fibrosis inhibition. Nevertheless, it is worth noting that the low water solubility of CUR presents challenges to its usage [26]. Optimized preparation technology and better understanding of the regulatory mechanism of the interaction between macrophages and BMSCs and the subsequent effects on bone tissue regeneration are crucial for the application of curcumin in bone regenerative medicine.

This study employs a simple and effective method to modify CUR on the surface of copper titanium metal in order to explore the effect of CUR on host bone immunomodulatory performance, and to explore the interaction between macrophages and BMSCs.

## 2. Results

### 2.1. Characterizations of CUR Modified Surfaces

**Figure 1:**
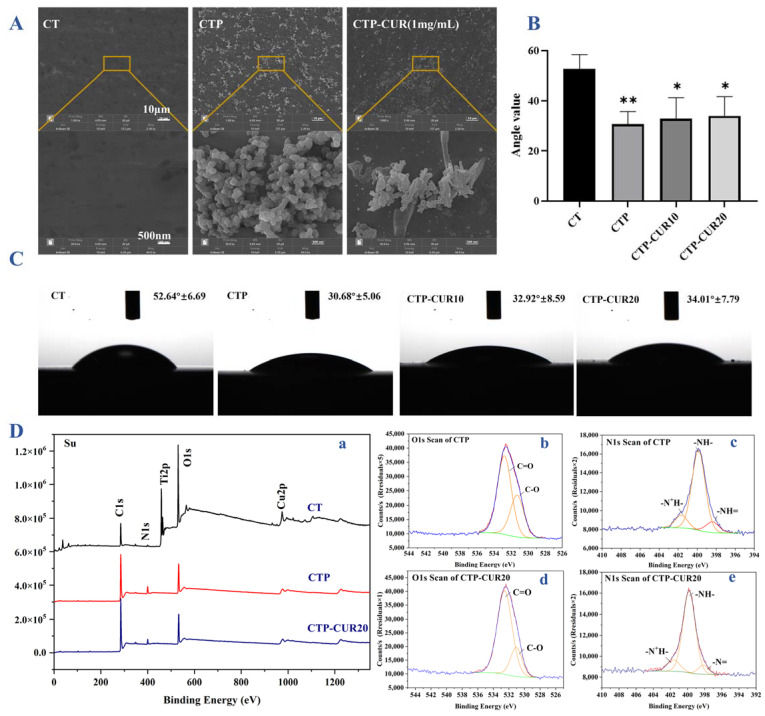
Characterizations of CUR modified surfaces: (**A**) surfaces topography detected by FE-SEM; (**B**) water contact angle of the samples; (**C**) wettability of the samples; (**D**) XPS spectrum of the samples. * *p* < 0.05 and ** *p* < 0.01 are the statistic difference other groups and CT.

In order to better understand the morphology of the modifications, we applied a higher concentration of CUR in field emission scanning electron microscope (FE-SEM) detection. As shown in Figure 1A, after polydopamine (PDA) modification, the surface of the Cu-Ti sample (named as ‘CT’) was covered with a uniform circular nanoparticle layer and several rod-shaped objects appeared as a CUR coating. The coating of both the PDA and CUR enhanced the wettability of the sample surface with a smaller water contact angle (Figure 1B,C). Furthermore, an X-ray photoelectron spectroscopy (XPS) test (Figure 1D) confirmed that the stable presence of CUR could be detected even at low concentrations of 20 μM in subsequent experiments. In terms of the pure copper-bearing samples, Ti and Cu were primarily present, and the N element appeared following the modification of PDA (Figure 1D(a)), which is the key component of dopamine. Upon attachment of CUR on the samples that the group of Cu-Ti modified with Polydopamine (named as ‘CTP’), the N content decreased due to the absence of N in CUR, while the C content increased because of the inclusion of N in CUR. In the fitting peaks showed in Figure 1D(b–e), 531.3 eV and 532.79 eV were C=O and C-O bonds respectively, while 398.4 eV, 399.86 eV and 401.67 eV corresponded to -N=, -NH- and -N^+^H-. In O 1s orbital, the C-O bond content increased from 65.84% to 78.56% after CUR modification, while the C=O bond content decreased from 34.16% to 21.44%, indicating that more C=O was reacted. In N 1 s, the content proportion of -NH- in the fitting figure increased from 77% to 80.7% after CUR modification, confirming that more N-related chemical bonds reacted. 

### 2.2. Osteogenic Capability of BMSCs on CUR Modified Surfaces

**Figure 2:**
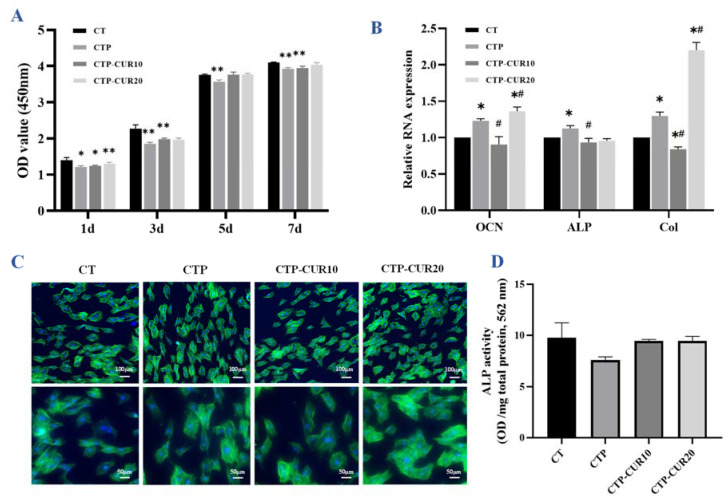
Osteogenic capability of BMSCs on CUR modified surfaces: (**A**) CCK8 assay for cell viability of BMSCs cultured on samples for 1, 3, 5 and 7 days; (**B**) expression of osteogenic genes in BMSCs after culture for 7 days; (**C**) cell adhesion situation and morphology of BMSCs on samples for 24 h; (**D**) ALP activity in BMSCs on samples after osteogenic induction for 7 days. * *p* < 0.05 and ** *p* < 0.01 are the statistic difference other groups and CT; # *p* < 0.05 is the statistic difference other groups and CTP.

The BMSCs had the highest cell ability when cultured on pure Cu-Ti samples for 1, 3, 5 and 7 days, while the BMSCs had grown well on the CTP group and experimental groups with all relative growth rates higher than 80%, and matched the ‘S’ growth curve (Figure 2A). The BMSC cells spread evenly on each group and adhered well (Figure 2B), which was consistent with the results of the cell viability experiment. The coating of CUR induced the expression of the osteogenesis genes osteocalcin (OCN), alkaline phosphatase (ALP) and type 1 collagen (COL-1), especially at the concentration of 20 μΜ (Figure 2C), while having no significant difference in terms of the effect of ALP activity (Figure 2D).

### 2.3. Immune Response of RAW Cells on CUR Modified Surfaces

**Figure 3:**
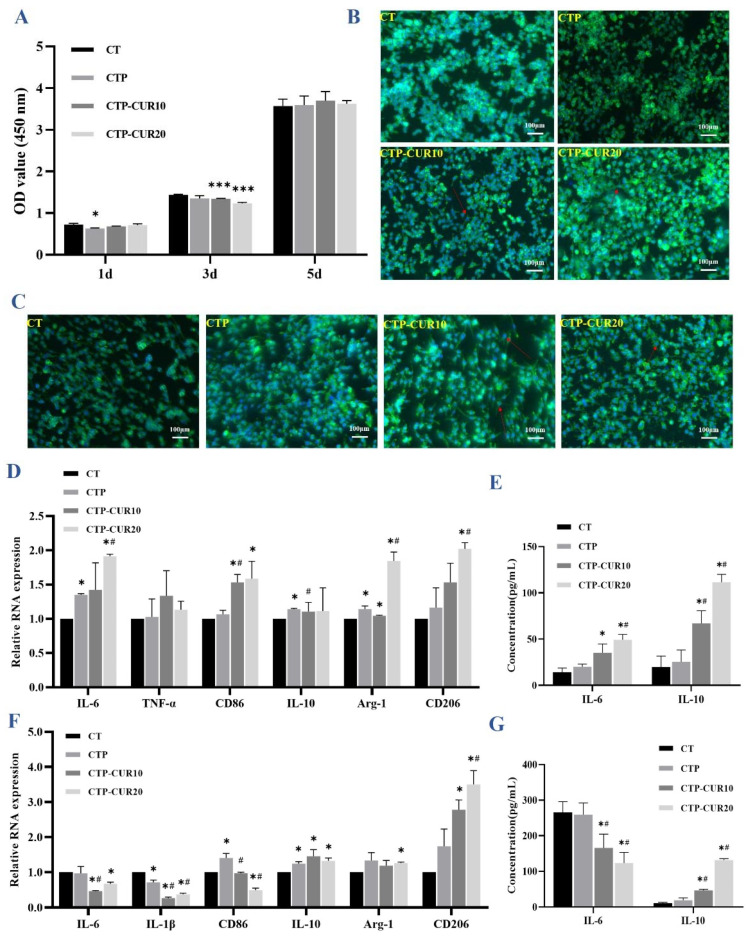
Inflammatory and anti-inflammatory response of RAW 264.7 on CUR modified surfaces: CCK8 assay for the cell viability of RAW cells cultured on samples for 1, 3 and 5 days (**A**); morphology of RAW cells on samples (24 h) under normal condition **(B**) and inflammatory condition (**C**); gene expression of inflammatory cytokines, anti-inflammatory cytokines and phenotype markers (CD86 and CD206) under normal condition (**D**) and inflammatory condition (**F**); concentration of IL-6 and IL-10 in the cell supernatant measured by ELISA under normal condition (**E**) and inflammatory condition (**G**). * *p* < 0.05 and *** *p* < 0.001 are the statistic difference other groups and CT; # *p* < 0.05 is the statistic difference other groups and CTP.

The cell activity assay showed that the coating of PDA and CUR had no toxicity on the RAW cells when cultured for 5 days, despite the optical density (OD) value being slightly lowered by the coatings on days 1 and 3 (Figure 3A). To verify the aforementioned result, the morphology of the RAW cells was detected by phalloidin-4′,6-Diamidino-2-Phenylindole (DAPI) staining. All groups had a similar density of RAW cells (Figure 3B, C). Under normal condition, the RAW cells were mostly round in shape with a few cells featuring antennas in the CUR-modified groups (Figure 3B). Under the inflammatory condition, most of the RAW cells were stimulated to form polygonal cells, and several cells were elongated axially in the CUR-modified groups (Figure 3C). Under the normal condition, the quantitative real-time PCR (RT-qPCR) results showed that the CUR modification up-regulated the expression of both inflammatory cytokine-related genes (interleukin-6 (IL-6) and tumor necrosis factor-α (TNF-α)) and anti-inflammatory genes (interleukin-10 (IL-10) and arginine-1 (Arg-1)), including genes of phenotypic markers CD86 and CD206 (Figure 3D). In the inflammatory condition, the expression of inflammatory genes and CD86 was reduced by CUR modification, while the expression of anti-inflammatory genes and CD206 was still elevated in the experimental groups (Figure 3F). The results of the cytokines IL-6 and IL-10 as measured by enzyme-linked immunosorbent assay (ELISA) were consistent with the results of RT-qPCR (Figure 3E,G).

### 2.4. Effect of Macrophage-Conditioned Medium (CM) in Normal Condition on the Osteogenesis of BMSCs

**Figure 4:**
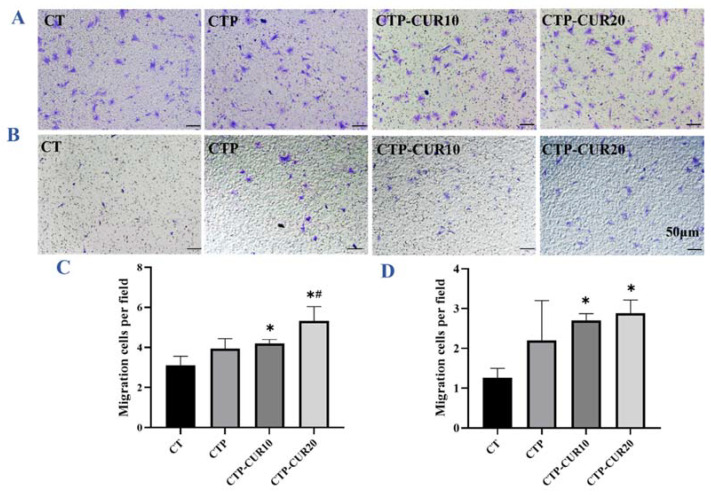
Migration of BMSCs cultured by macrophage-CM for 24 h on CUR modified surfaces: images and quantitative number of migrated BMSCs in the normal state (**A**,**C**) and the inflammatory state (**B**,**D**). * *p* < 0.05 is the statistic difference other groups and CT; # *p* < 0.05 is the statistic difference other groups and CTP.

**Figure 5:**
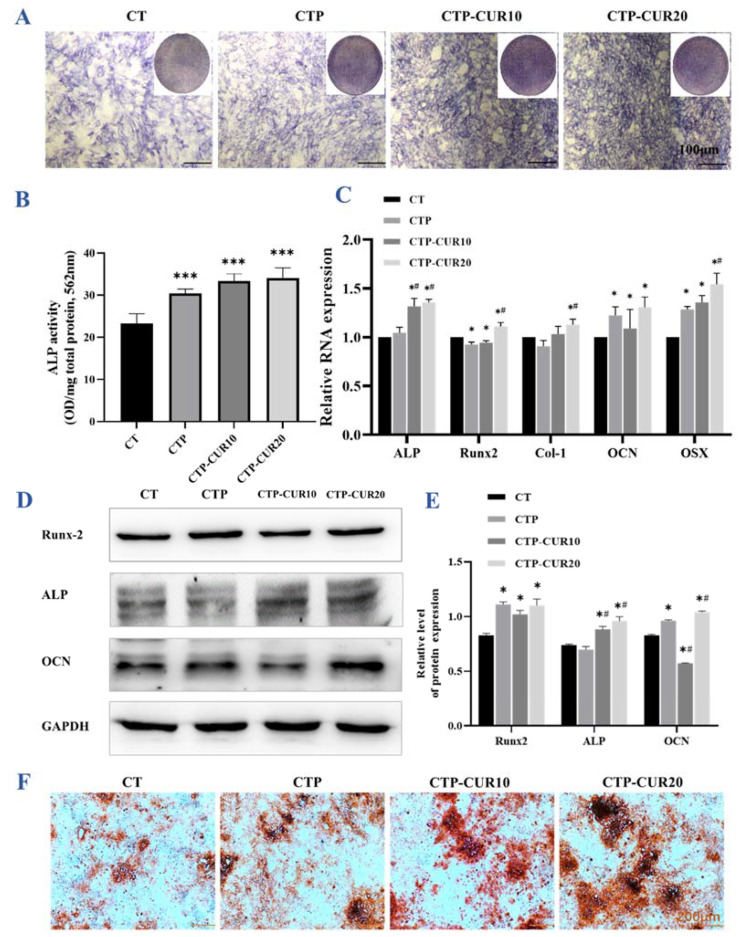
Immunomodulatory effects of macrophage-CM under normal condition of BMSCs on CUR modified surfaces: (**A**,**B**) ALP staining images and quantitative analysis of BMSCs cultured by CM for 7 days; (**C**) relative gene expression of osteogenic genes in BMSCs induced by CM for 7 days; Western blot images (**D**) and quantitative expression (**E**) of osteogenic-associated proteins in BMSCs stimulated by CM for 7 days; (**F**) mineralized nodules of BMSCs stained with ARS on day 14. * *p* < 0.05; *** *p* < 0.001 are the statistic difference other groups and CT; # *p* < 0.05 is the statistic difference other groups and CTP.

Under the normal condition, the BMSCs migrated well (Figure 4A) compared to those in the inflammatory condition (Figure 4B). The migration images reveal that the coating of PDA and CUR, respectively, induced more BMSCs to be successfully migrated through the transwell chamber (Figure 4A). The quantitative calculation results accorded with those of the migration (Figure 4C). ALP staining (Figure 5A) and the quantitative results (Figure 5B) corresponded to each other. Briefly, the coating of PDA and CUR at 10 μΜ increased the ALP activity slightly, while CUR at 20 μΜ had more obvious effects. The expression of osteogenesis genes was quantified by RT-qPCR (Figure 5C). CUR modification at the concentration of 20 μΜ improved the expression of all genes mentioned (ALP, runt-related transcription factor 2 (Runx-2), COL-1, OCN and Osterix (OSX)), and all of these were statistically different from CTP, except COL-1. CUR modification at the concentration of 10 μΜ only upgraded the expression of ALP, Runx-2, OCN and OSX, with no significances from the positive control group CTP. The Western blot chemiluminescence band results and gray value ratio results show that the group with CUR modification at 20 μΜ increased the expression of osteogenesis proteins (ALP, Runx-2 and OCN) (Figure 5D,E). In contrast, CUR modification at 10 μΜ had the opposite effect on the Runx-2 and OCN proteins, despite having a positive effect on ALP (Figure 5D,E). The Alizarin Red S (ARS) staining results indicated that CUR modification improved the mineralization ability of BMSCs (Figure 5F).

### 2.5. Effect of Macrophage-CM in Inflammatory Condition on the Osteogenesis of BMSCs

**Figure 6:**
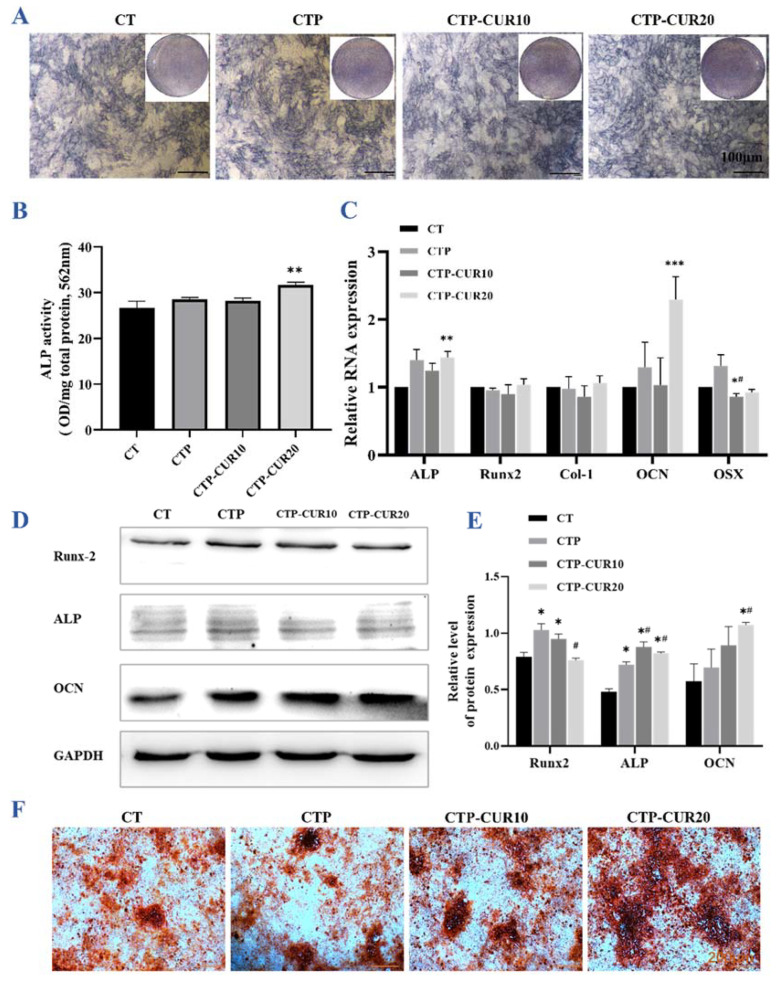
Immunomodulatory effects of macrophage-CM under inflammatory condition of BMSCs on CUR modified surfaces: (**A**,**B**) ALP staining images and quantitative analysis of BMSCs cultured by CM for 7 days; (**C**) relative gene expression of osteogenic genes in BMSCs induced by CM for 7 days; (**D**,**E**) Western blot images and quantitative expression of osteogenic-associated proteins in BMSCs stimulated by CM for 7 days; (**F**) mineralized nodules of BMSCs stained with ARS on day 14. * *p* < 0.05, ** *p* < 0.01 and *** *p* < 0.001 are the statistic difference other groups and CT; # *p* < 0.05 is the statistic difference other groups and CTP.

In the inflammatory state, only a small number of BMSCs cultured by CM migrated in each group, but the CUR coating had a probable positive effect (Figure 4B,D). In terms of ALP staining and quantification, only the group with the 20 μΜ CUR modification had a different result compared to the control group (Figure 6A,B). The RT-qPCR result had a similar tendency, and only the modification of CUR at 20 μΜ increased the expression of osteogenesis genes (ALP and OCN), with no significant difference from the CTP group (Figure 6C). However, the coating of both PDA and CUR up-regulated the expression of osteogenesis proteins (Runx-2, ALP and OCN), although there was no significant difference from the control groups of gene OCN (Figure 6D,E). The ARS staining results show that CUR modification increased the mineralization of BMSCs (Figure 6F).

## 3. Discussion

CUR is insoluble in water, but soluble in ethanol, acetone, dimethylsulfoxide (DMSO) and other solvents [26]. These chemical solvents limit the in vivo application of CUR in relation to their biocompatibility. As mentioned above, implant surface modification is currently a highly recommended method, among which PDA is an efficient and simple intermediate modification method. PDA can adhere to almost all types of substances and can be utilized as an anchor layer to graft different materials, which is a commonly employed approach in implant surface modification [6,27]. For example, graphene oxide [28], Ag ions [29] and polypeptides [30] can be successfully modified to perform biological functions on an implant surface. As a Chinese herbal medicine, CUR has been proved to be able to bind with PDA, and can even be modified on the surface of pure titanium by hydrogen bonding [18]. In our study, we clearly saw CUR modified onto the Cu-Ti surface by PDA (Figure 1A). Additionally, even a very low concentration of CUR, which was considered as having no toxic in our following biological experiments, could be detected by XPS. After CUR modification, the proportion of the N element content in PDA decreased, confirming that CUR was modified on the surface of the metal sample (Figure 1D). This was consistent with the FE-SEM results—PDA was a circular nanoparticle polymer, while CUR was a rod-shaped powder (Figure 1A). 

In addition to using applicable solvents, nanocarriers can be used to improve the utilization rate of CUR, but slow down the release rate of CUR [31]. Nevertheless, chemical grafting and other modification methods can effectively improve the availability of CUR [32]. In our experiment, through the strong and stable connection of PDA, CUR was fixed onto the metal surface, which was supposed to play a direct and long-term effect on the tissue-metal interface. However, due to the current concentration limitation and the structure characteristics of micron CUR, the surface bonding amount of CUR was restricted. Nano-curcumin could perhaps further improve this issue.

The water contact angle on the surface of the implant is the main factor of biocompatibility, while improved hydrophilicity is conducive to cell adhesion [33]. In this study, the modification of PDA and CUR reduced the water contact angle on the sample surfaces. CUR is mainly composed of unsaturated lipids and aromatic groups, resulting in its low water solubility [31]. Accordingly, CUR and the increase of CUR concentration weakened the surface affinity slightly, but it was still significantly better than that of a pure Cu-Ti surface (Figure 1B,C). This was perhaps related to the decrease of the water contact angle with the nanoscale surface modifications and the increase of surface roughness. The above results were correlated with the cellular adhesion to some extent. As shown in Figure 2B and Figure 3C, RAW 264.7 and BMSCs had good adhesion in modified groups, indicating that the surface of Cu-Ti and the modification of PDA/PDA-CUR had no toxicity for cell growth. Yang’s group has revealed that the relative growth rate of BMSCs on Cu-Ti was higher than 100%, compared to that on a pure titanium sample [4]. In this study, on the one hand, CUR modification slightly reduced the cell viability of RAW 264.7 and BMSCs, with the acceptable relative growth rate higher than 80%, considering that CUR and PDA modification have no toxicity according to the ISO 10993-5 standard [34]. In addition, CUR also has no toxicity to gingival fibroblasts in an in vitro experiment [35]. On the other hand, CUR had no significant positive effect on cell adhesion, which may be related to the heterogeneity of the current modification. Remarkably, CUR (20 μM) in the CTP groups could synergistically promote the expression of OCN and COL-1 with PDA nanoparticles (Figure 2C), although there was no statistically significant difference in ALP activity (Figure 2D). Furthermore, the CUR/PDA synergistic effect in this study appeared to be concentration-dependent as there was no promotion at 10 μM. Studies have showed that CUR inhibits the osteogenesis of BMSCs [36], but other research has indicated that the application of CUR enhanced osteoblastic migration during BMSCs therapy [37,38]. In our study, modification of CUR (20 μM) and PDA nanoparticles showed a slight promotion effect on BMSCs, perhaps due to the low concentration, but definitely did not reduce the osteogenic effect of Cu-Ti on BMSCs.

Macrophages in the host could differentiate in response to changes in the microenvironment, which could be validated both at the cellular and molecular levels. Generally, with an increase in cell pseudopods [39,40], M1 macrophages primarily mediate the occurrence of inflammation and express inflammatory cytokines interleukin-1β (IL-1β), IL-6, TNF-α and surface marker CD86 [13,41]. With axial elongation of cells [39,40], M2 macrophages could inhibit inflammation, while promoting angiogenesis, wound healing and tissue fibrosis, and expressing anti-inflammatory factors Arg-1, IL-10 and surface marker CD206 [41].

Whether the Cu-Ti alloys could influence the immunomodulatory process is still unclear. In the physiological and inflammatory state, more macrophage cells were activated to grow evident pseudopods in both the CUR-CTP10 and CUR-CTP20 groups (Figure 3B,C), in which Cu-Ti and PDA nanoparticles might have no influence. Besides, CUR modification could induce the differentiation of M1-type cells (pro-inflammation) in the physiological state and increase of M2-type cells (anti-inflammation) in the inflammatory state, illustrating that the participation of CUR could regulate the polar differentiation of macrophages, thus improving bone healing. The results were consistent with a previous study [42], which indicates that CUR significantly reduced the ratio of M1:M2, thus controlling the occurrence of diseases. This phenomenon might be due to CUR activating IκBα to inhibit the expression of downstream signals of the NF-κB pathway, and then inhibiting the polarization of macrophages from the M0 to M1 phenotype [43]. Additionally, it has been proved that CUR could activate the signal transducer and activator of the transcription 6 (STAT6)/PPAR-γ signaling pathway to induce the polarization of macrophages from uncommitted macrophages to the M2 phenotype, or from the M1 to the M2 phenotype [43,44]. Therefore, with the influence of polarization, surface molecule expression, cytokine and chemokine production and their underlying pathways of macrophages, CUR controls mycobacterium infection, obesity, atherosclerosis, cancer, etc. [42]. Subsequent molecular experiments also confirmed these results. Under the physiological state in our study, the modification of CUR simultaneously induced the high expression of inflammatory genes and cytokines in the M1 and M2 phenotypes (Figure 3D,E), whereas these changes were not significant in the CT or CTP groups. These outcomes indicated that there was no evidence that Cu-Ti or PDA nanoparticles had effects on the macrophages under the normal physiological condition, and only the addition of CUR could significantly activate cell polarization, which resembled the phlegmonosis status. Furthermore, the expression of IL-6 increased with the concentration of modified CUR (Figure 3E), which has also been found in Loo’s study [45], and the discrepancy might cause by the diversity of surface charge of CUR. Additionally, in the physiological state, CUR modification inducing uncommitted macrophages that became polarized toward a M1 phenotype might also explain the increased expression of IL-6. These macrophages switche to the M2 phenotype at later stages to promote bone repair by producing growth factors to recruit BMSCs and guide the osteo-differentiation (Figure 6). CUR modified Cu-Ti can suppress inflammation and promote healing, which was confirmed in our subsequent results. However, the specific mechanism by which CUR up-regulates IL-6 expression in normal conditions need further exploration. In the inflammatory state, the modification of CUR effectively inhibited the expression of IL-6, IL-1β and CD86, while inducing the expression of IL-10, Arg-1 and CD206 (Figure 3F), which was in line with the ELISA results (Figure 3G). As the main immune cells, macrophages are very important for the postoperative effect of dental implantation. According to the above experimental results, in general, CUR modification improved the bone immune regulation of Cu-Ti through directly regulating the polar differentiation of macrophages and, more specifically, down-regulating the expression of M1, and up-regulating the expression of M2. It is suggested that the modification of CUR may also be beneficial to inflammatory control and tissue repair after implantation.

Despite a direct effect on macrophages, CUR was expected to regulate osteogenesis by the cells’ immune response. First of all, an appropriate amount of M1-type inflammatory factors such as IL-6 can induce the recruitment and migration of mesenchymal stem cells (MSCs) [46,47,48,49,50,51,52]. MSCs are multidirectional cells with the ability to self-renew and differentiate into a variety of cell types, thus playing a key role in tissue repair and regenerative medicine [49]. Under normal conditions, the modification of CUR induced more BMSCs migration in the CTP-CUR10 and CTP-CUR20 groups (Figure 4A,C), which may be related to the high expression of IL-6 and TNF-α as mentioned above [37,38,39,40,41,42,46,47,48]. Secondly, macrophages could regulate the osteogenic behavior of MSCs [12,50], while exosomes of BMSCs could regulate inflammation in the process of tissue healing, and promote the formation of fibrocartilage by increasing the polarization of M2 macrophages in the tendon-bone healing process, thereby improving the biomechanical properties [51,52]. Finally, CUR could be involved in the mutual regulation of macrophages and BMSCs through direct or indirect effects [29]. In our studies, the activity of osteogenic-related genes and proteins and the ALP of BMSCs were increased significantly under normal conditions. Research has showed that even low-dose TNF-α can improve bone formation [53], while IL-6 deficiency could impact on bone formation [54]. Therefore, IL-6 and TNF-α may cooperate with M2-type macrophages to promote the osteogenesis of BMSCs. In the state of inflammation, the overexpression of inflammatory factors such as IL-6 and IL-1β may be inhibited to control the overreaction of inflammation. In other words, the modification of CUR with PDA affected the behavior of osteoblasts by regulating the cytokine expression of macrophages in different states, and this might further improve the osteogenesis of Cu-Ti in both early implantation and inflammatory conditions. 

## 4. Materials and Methods

### 4.1. Principal Reagents

Ti-5 wt.%Cu alloy was kindly provided by Dr. Ke Yang (Institute of Metal Research, Chinese Academy of Sciences); CUR, dopamine hydrochloride and lipopolysaccharide (from *Escherichia coli* O127:B8) were purchased from Sigma-Aldrich (St. Louis, MO, USA); RAW 264.7 (TIB-71) was purchased from the global bioresource center (ATCC, TIB-71); BMSCs were isolated and cultured from Sprague-Dawley rats (Guangzhou, China); Dulbecco’s modified eagle medium (DMEM) was purchased from Giboco (New York, NY, USA); fetal bovine serum (FBS) was purchased from Corning (New York, NY, USA); DAPI was purchased from Solarbio (Beijing, China); an F-actin staining kit was purchased from Abbkine (Wuhan, China); ELISA kits were purchased from Elabscience (Wuhan, China); an ALP assay kit was purchased from Fdbio (Hangzhou, China); an 5-bromo-4-chloro-3-indolyl-phosphate/Nitro-Blue-Tetrazolium (BCIP/NBT) ALP staining kit was purchased from Beyotime Biotechnology (Shanghai, China); and primary rabbit antibodies and secondary antibody (anti-rabbit) were purchased from Abcam (Shanghai, China).

### 4.2. Sample Preparation and Characterization

Ti-5 wt.%Cu titanium alloys (diameter: 10 mm, thickness: 2 mm) were polished with silicon carbide paper (400#~7000#), and then ultrasonically cleaned with acetone, ethanol and deionized water. The modification of CUR onto the Cu-Ti surface was prepared by using a method similar to that of He et al., 2015 [18]; after drying and disinfection by ultraviolet light, the samples were immersed in dopamine solution (2 mg/mL) in Tris-HCl (10 mM, pH = 8.5) overnight in the dark. Then, CTP samples were washed with ultra-pure water and dried in a vacuum chamber, and furtherly immersed in CUR aqueous solution (1mg/mL for FE-SEM and 10 μM, 20 μM for other experiments) for 24 h. Subsequently, the samples were thoroughly rinsed with water, air-dried and respectively named as ‘CTP-CUR10′ and ‘CTP-CUR20′. The surface morphology was characterized by FE-SEM (Hitachi, 12kV, Tokyo, Japan), while the surface chemical compositions of the samples were verified by XPS (ThermoFish, Shanghai, China) through full XPS spectra and detailed spectra of the C 1s/N 1s/O 1s peaks. Moreover, the water contact angle was detected to determine the hydrophily of the samples.

### 4.3. Osteogenic Capability of the CUR Coating

#### 4.3.1. Isolation and Culture of BMSCs 

Bone marrow tissue was obtained from Sprague-Dawley rats (4-week-old males), and the relevant animal source was certified for quality and ethical approval. After centrifugation, the cells were incubated in DMEM with 10% (*v*/*v*) FBS in incubators with 5% CO_2_ at 37 °C. When the cell density was 80–90% of the dish, subculture was carried out and 3 to 5 generations were taken for subsequent experiments. The BMSCs were seeded at a density of 1 × 10^4^ cells/cm^2^ on the samples, and at 2 × 10^4^ cells/cm^2^ for the osteogenic differentiation experiments, which were induced with an osteogenic induction medium after 24 h on the samples.

#### 4.3.2. Cell Viability Test of BMSCs

After the BMSCs cells had been cultured on the samples for 1, 3, 5 and 7 days, the culture supernate was removed and replaced by fresh medium with 10% (*v*/*v*) CCK8 reagent (Meilun, Dalian, China). Following 4 h of incubation, the absorbance of the medium was tested 3 times at 450 nm, and the average value was obtained.

#### 4.3.3. Adhesion of BMSCs 

The adhesion condition and morphology of the BMSCs on samples were observed by cytoskeletal staining with phalloidin for 30 min and nuclear staining with DAPI for 5 min. After 24 h of culture on the samples, the medium was replaced by moderate 4% paraformaldehyde (PFA) (Leagene, Beijing, China) to fix the cells. Then, 1% Triton-X100 (Leagene, Beijing, China) solution was applied to increase the cell permeability prior to fluorescence staining. Triple 3 min flushing with PBS was performed between every two steps. Images were taken using an upright microscope (Olympus, Tokyo, Japan) and adjusted with ImageJ software.

#### 4.3.4. ALP Activity Assay

After osteogenic induction for 7 days, the BMSCs were rinsed with PBS 3 times and lysed with 1% Triton-X100 on ice. Following centrifugation for 15 min (12000 rpm, 4 °C), the supernatants were collected for ALP activity testing with an ALP assay kit, while the total protein concentration was measured with a bicinchoninic acid (BCA) protein assay kit (Fdbio, Hangzhou, China). The ALP levels were normalized to the total protein concentration.

#### 4.3.5. Osteogenic Gene Expression

After osteogenic induction for 7 days, the BMSCs were lysed with RNAiso (Takala, Shiga, Japan) to obtain the total RNA. After measuring the RNA concentration using a NanoDrop ND-1000 spectrophotometer (Gene, Shanghai, China), 1000 ng of total RNA was applied to synthesize complementary DNA (cDNA). Then, this cDNA was united with TBGreen qPCR Master Mix (Takala, Shiga, Japan) to quantify the expression of osteogenesis genes by RT-qPCR (LightCycler480, Roche, Germany). The targeted genes included COL-1, ALP, Runx-2, OCN and glyceraldehyde-3-phosphate dehydrogenase (GAPDH), with the latter identified as a housekeeping gene. The relative gene expression level was achieved by utilizing the comparative 2^−ΔΔCt^ method.

### 4.4. Macrophage Polarization and Inflammatory Response to CUR Coating 

#### 4.4.1. Cell Culture of RAW 264.7

The RAW 264.7 cell line was employed to investigate the effect of CUR coating on macrophage polarization and the inflammatory response in normal and inflammatory situations. RAW cells were cultured in DMEM with 10% (*v*/*v*) FBS in incubators with 5% CO_2_ at 37 °C. Normally, the RAW cells were seeded in 24-well plates with a density of 2 × 10^5^ cells/cm^2^, which was the case except for special experiments.

Under the normal condition, RAW cells were cultured on different samples for 24 h and the medium (named CM) was collected for the subsequent experiments. For the inflammatory condition, DMEM medium with 1 μg/mL lipopolysaccharide was utilized to stimulate RAW cells for 2 h after 12 h culture on the samples, and finally replaced by serum-free medium for 6 h. The supernatant was also collected for CM usage. All CM was filtered with 0.2 nm filters following centrifugation for 10 min (1000 rcf, 4 °C), and stored at −80 °C in a refrigerator. 

#### 4.4.2. Cell Viability of RAW 264.7

The RAW cells were seeded on samples at a density of 2 × 10^4^ cells/cm^2^ and the cell viability was evaluated by CCK8 on days 1, 3 and 5, as detailed in Section 4.3.2.

#### 4.4.3. Cell Morphology of RAW 264.7

The RAW cells were fixed with 4% PFA and lysed with 1% Triton-X100 after culture on samples for 24 h, with the phalloidin-DAPI staining procedure detailed in Section 4.3.3.

#### 4.4.4. Inflammatory Gene Expression

The RT-qPCR operative details were as described in Section 4.3.5. Both the inflammatory genes and anti-inflammatory genes were quantified. The former included IL-6, IL-1β and TNF-α, while the latter comprised IL-10 and Arg-1. The genes of the cell surface markers (CD86 and CD206) were also measured.

#### 4.4.5. Expression of Inflammatory Cytokines

The expression of inflammatory cytokines was measured using an ELISA kit, with the supernatant collected and tested according to the manufacturer’s instructions. According to the standard curve, the secretion volumes of TNF-α and IL-10 were calculated.

### 4.5. Osteoimmunomodulatory Effect of CM from Macrophage

All the CM mentioned above was thawed and mixed with normal osteogenesis induction medium at a ratio of 1:1. After seeding for 24 h, the BMSCs were cultured in the commixture medium, during which the medium was refreshed every 2 to 3 days.

#### 4.5.1. Migration Capacity of BMSCs

Transwell chambers (Corning, New York, NY, USA) were employed to evaluate the migration capacity of the BMSCs. In the inner chamber, 1 × 10^4^ BMSCs were seeded with an 8.0 μm pore size and cultured for 12 h. Then, commixture medium of different groups was applied to the bottom of the chamber in the 24-well plate and cultured for a further 12 h. After removing the cells without migration, those cells with successful migration on the underside of the chamber were fixed with 4% PFA and then stained with 1% crystal violet solution (Leagene, Beijing, China). The migration amount of each transwell chamber was calculated by counting the cells in 5 random views, examined under a microscope (Olympus IX53, Tokyo, Japan).

#### 4.5.2. Expression of Osteogenesis Protein 

The total proteins of the BMSCs were purified with lysis solution, centrifuged, electrophoresed, and then passed on to polyvinylidene difluoride (PVDF) membranes. After blocking in quick-block solution (Beyotime, Zhejiang, China), all PVDF membranes were incubated overnight in primary antibody at 4 °C. Secondary antibody incubation was performed in the next stage, and finally all membranes were displayed by enhanced chemiluminescence to reveal the protein band. The expression of protein was measured by the gray value of each band, processed by ImageJ software.

#### 4.5.3. ALP Activity Assay

The methods for quantitative detection of the ALP were as detailed in Section 4.3.4. ALP staining was performed with a BCIP/NBT staining kit. Briefly, after culturing by CM for 7 days, the BMSCs were rinsed with PBS and fixed with 4% PFA, and then stained with the kit and observed by stereo microscope (Olympus, Tokyo, Japan).

#### 4.5.4. Mineralization of BMSCs

The mineralization capacity of the BMSCs cultured by CM was analyzed utilizing 2% ARS staining. The BMSCs were cultured for 14 days, after which the cells were fixed and stained with ARS solution. Subsequently, the staining images of the BMSCs were captured by microscope.

### 4.6. Statistical Analysis

All experiments were repeated 3 times, with all the data shown as the mean ± standard deviation. GraphPad Prism 8.0 software was employed to carry out the statistical analysis. Differences between groups were analyzed via one-way analysis of variance (ANOVA), followed by the Tukey post-hoc test. Differences were considered statistically significant when *p* < 0.05.

## 5. Conclusions

CUR can be modified onto the surface of copper-bearing titanium with PDA by a simple and effective method. CUR concentrations of 10 μM and 20 μM on Cu-Ti could regulate the polar differentiation of macrophages in both normal and inflammatory conditions, and therefore promote the osteogenic effect of BMSCs through the immune regulation of macrophages. Further studies should be done to optimize the technology and explore the causative mechanism.

## Data Availability

All data contained within this article.

## Sample Availability

Samples of the compounds are not available from the authors.
